# Development of New Efficient Adsorbent by Functionalization of Mg_3_Al-LDH with Methyl Trialkyl Ammonium Chloride Ionic Liquid

**DOI:** 10.3390/molecules26237384

**Published:** 2021-12-05

**Authors:** Samuel Nick Țolea, Laura Cocheci, Lavinia Lupa, Raluca Vodă, Rodica Pode

**Affiliations:** Faculty of Industrial Chemistry and Environmental Engineering, University Politehnica Timisoara, Vasile Parvan Blv. No. 6, 300223 Timisoara, Romania; samy_nick2008@yahoo.com (S.N.Ț.); raluca.voda@upt.ro (R.V.); rodica.pode@upt.ro (R.P.)

**Keywords:** layered double hydroxides, ionic liquid, functionalization, adsorption, diclofenac

## Abstract

The present paper describes a new way of obtaining an efficient adsorbent material by functionalization of Mg_3_Al layered double hydroxides (LDH) with methyl trialkyl ammonium chloride-ionic liquid (IL) using two methods: ultrasound and cosynthesis. Layered double hydroxides are good solid support for the functionalization with ionic liquids due to their well-ordered structure. The immobilization of the ILs in suitable solid supports combine the advantages of the ILs with the properties of the solid supports bringing more benefits such as use of lower quantity of ILs and avoiding of ILs loss in the aqua phase which overall decrease the treatment costs. In case of ultrasound method of functionalization is assured a uniform distribution of IL on the solid surface, but through immobilization by cosynthesis due to the tunable properties of LDH, is assured an intercalation of the ILs between the LDH layers. This fact was highlighted by the X-ray diffraction (RXD), scanning electron microscopy (SEM) analyses and Fourier-transform infrared (FTIR) spectroscopy of the obtained adsorbent. The added value brought by the functionalization of Mg_3_Al with the studied IL was underlined by the adsorption studies conducted in the treatment process of water with diclofenac content. Kinetic, thermodynamic, and equilibrium studies were performed. DCF adsorption onto the studied materials correspond to a chemisorption, the pseudo-second-order kinetic model describing the most accurately the experimental data. DCF adsorption onto the studied materials occurs as a heterogeneous process, with the experimental data fitting best with the SIPS isotherm. The sample obtained through cosynthesis developed a maximum adsorption capacity of 648 mg/g.

## 1. Introduction

Water is an essential nutrient used in many fields, and for this reason keeping the water sources as fresh as possible, by pollution prevention or by development of efficient method of treatment, is the main concern of humanity [1]. Water treatment technologies were intensively studied, and after a primary treatment, it is more often necessary to use an advanced water treatment technology. This is especially the case when it is necessary to remove trace contaminants, which, even if they are found in low concentrations, are very harmful for living organisms such as: heavy metals and drugs. From the studied advanced water treatment technologies, adsorption is one of the most efficient due to its ease of operation and high-performance results [2,3,4]. The most important factor in the adsorption processes to obtain great efficiencies is represented by the used adsorbent materials. Therefore, the development of new efficient adsorbent materials is still a challenge. An efficient adsorbent material must meet the following requirements: high adsorption kinetics and capacity [5], excellent selectivity [3], great thermal and chemical stability [4,6], and ease of synthesis [5,7].

These properties are fulfilled by layered double hydroxides (LDH). Layered double hydroxides (general formula—[MII_1−x_MIII_x_ (OH)_2_]^x+^ [A^n−^_x/n_ ^.^ mH_2_O]^x−^, where MII is a divalent cation, MIII is a trivalent cation and A^n−^ is an anion) are inorganic layered compounds containing positively layered surface of mixed metal hydroxides, which are balanced by the anions present in the interlayer region. Layered double hydroxide compounds are easily synthesized, present good high-specific surface area, and develop memory effect and ion exchange properties. Compared with that of the natural clays, the LDH compounds can be synthesized to the desired form using a wide range of chemical composition. Their structure can be modified by impregnation, intercalation, and/or functionalization, leading to improved properties. Using the synthetic route, LDHs can be obtained with various particle sizes. They possess higher layer charge density, and due to their intercalation ability, they develop stronger electrostatic forces, so they could be used as storage matrix or anionic exchangers [6,7]. Therefore, these materials represent significantly potential adsorbents [8,9,10,11].

In recent years, researchers focused on the functionalization of various materials to enhance their adsorbent properties [3,12,13]. In this direction, the use of ionic liquids (ILs) represents an efficient alternative due to their well-known advantages: good physical properties including high thermal stability, viscosity, density, lower solubility in water, and lower vapor pressure [14,15]. Considering their properties, ILs were used in many fields such as gas separations [16], metal extractions [17,18], drug delivery [19], and wastewater treatment [15]. ILs were intensively used in the liquid–liquid extraction processes, but lately they showed superior performance when mobilized on suitable solid supports and used for aqueous solutions treatments. Using ionic liquid-modified solid supports as new adsorbent materials, the draw backs of liquid–liquid extraction are avoided. In these processes, a smaller quantity of ILs is used and their loss in the aqueous phase is avoided; therefore, the overall costs are minimized [12,17,18,20,21].

Considering the highly tunable interior structure of LDH and the advantages brought by the ILs, in the present paper a new adsorbent material by functionalization of Mg_3_Al—LDH with methyl trialkyl ammonium chloride ionic liquid was developed. The methyl trialkyl ammonium chloride was chosen to functionalize the Mg_3_Al-LDH due to: (i) its insolubility in water, in this way avoiding its loss in the aqueous phase; (ii) the presence of three long alkyl chains (C8, C9, C10), which also could represent some potential sites for physical adsorption; and (iii) its carrier ability, conferred by its active sites, NH_4_^+^ and Cl^−^. The immobilization of the ILs in suitable solid supports combine the advantages of the ILs with the properties of the solid supports [22,23]. To highlight its improved adsorption properties, the obtained adsorbent materials were used in the treatment process of waters with diclofenac (DCF) content, a nonsteroidal, anti-inflammatory pharmaceutical drug. This is frequently used for treatment of different diseases, and its large consumption led to its detection in various water resources and wastewater treatment plant effluents [24,25]. This compound generates negative environmental impacts and serious health side-effects, and therefore, the removal of DCF from wastewater and polluted water is imperative [26,27,28].

## 2. Results and Discussion

### 2.1. Adsorbent Materials Characterization

**Figure 1:**
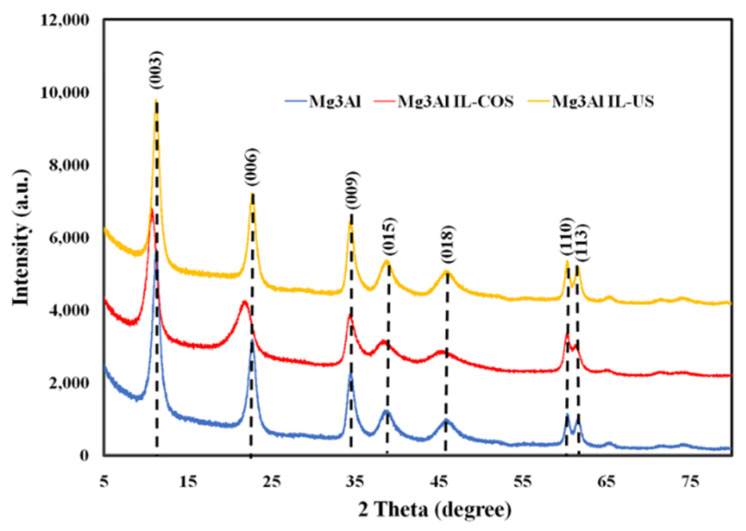
X-ray diffraction (XRD) patterns of synthesized samples.

The RX diffractograms of the synthesized Mg_3_Al raw material and of the ultrasonication and cosynthesis functionalized LDH (Mg_3_Al IL-US and Mg_3_Al IL-COS, respectively) are presented in Figure 1, and the lattice parameters and the crystallites size are shown in Table 1.

All the three adsorbent materials present the only crystalline phase those of the layered double hydroxide. The crystallinity of Mg_3_Al and Mg_3_Al IL-US samples is evidenced by the characteristic peaks corresponding to (003) and (006) planes (at low values of 2*θ* angles) and corresponding to (110) and (113) planes (at higher values of 2*θ* angles), which are sharp and symmetrical [29]. The ultrasound method of functionalization with the studied IL does not affect the crystallinity of Mg_3_Al IL-US sample. In case of Mg_3_Al IL-COS, this well-ordered structure is disturbed because, in this case, the IL is not just attached on the surface of Mg_3_Al, it could be found in the interlayer space, leading to a decrease in the sample crystallinity, and therefore, a more amorphous sample is obtained. In this case, the corresponding peaks to (003) and (006) planes are shifted to lower 2*θ* angles, presenting a flattering of the maxima/a lower intensity. The data presented in Table 1 show that the interlayer space increases from 23.58 to 24.39 Å, leading to a decrease in the crystallite size from 4.86 to 3.38 nm. These results prove the intercalation of the IL between the LDH layers [14,22,30]. In contrast, in the case of the Mg_3_Al IL-US sample, the distance between the layers does not show a significant change, indicating the functionalization with IL by ultrasound at the surface of the adsorbent.

**Figure 2:**
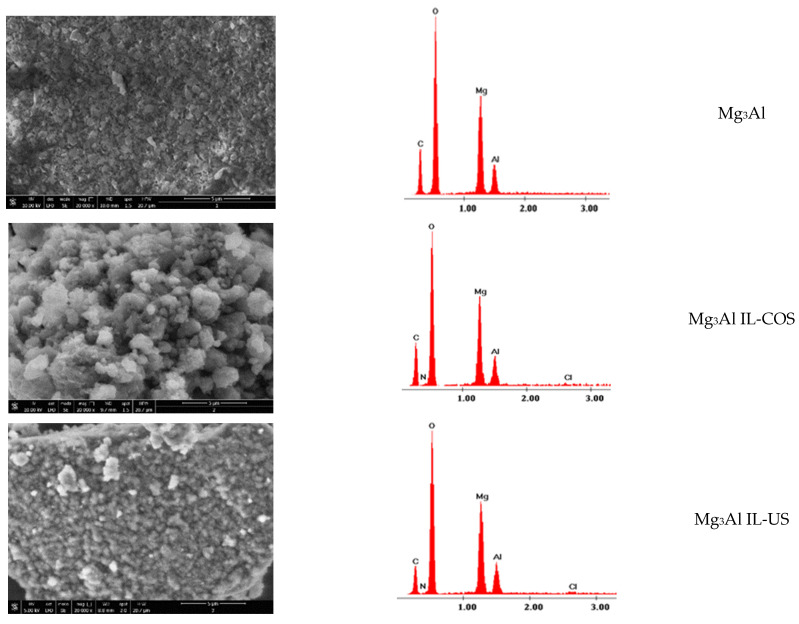
SEM images (at a magnification of 20,000×) and EDX spectra of studied adsorbent materials.

Scanning electron microscopy (SEM) images and Energy Dispersive X-ray Spectroscopy (EDX) spectra of studied adsorbent materials are shown in Figure 2.

The morphology of the Mg_3_Al sample is presented in the form of hexagons arranged in order in overlapping layers, specific to layered double hydroxides [8]. The sample functionalized by cosynthesis shows a network change: the layers are disordered due to the intercalation from place to place of the ionic liquid between LDH layers. The sample surface is presented like cotton flowers. For the sample functionalized by ultrasound method, this network disorder does not occur, as only the attachment of the ionic liquid to the surface of the solid support is observed, which leads to its homogenization. Mg_3_Al functionalization with the studied IL was also highlighted by EDX analysis in which the presence of nitrogen and chlorine could be observed.

### 2.2. Adsorption Studies

#### 2.2.1. pH Influence upon the Adsorption Capacity Developed by the Studied Materials

**Figure 3:**
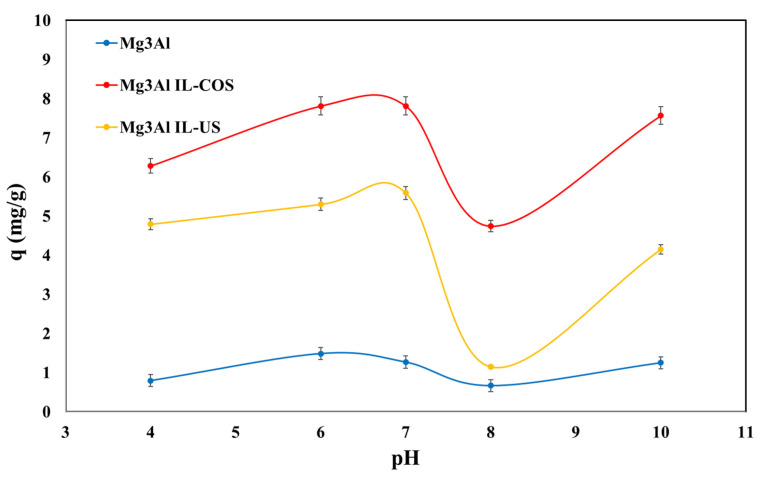
pH influence upon adsorption capacity developed by studied materials in removal process of diclofenac adsorption (DCF) from aqueous solutions.

In adsorption processes, the initial pH of the solutions plays an important role since it influences the surface of the adsorbent. The experimental results on the study of diclofenac adsorption (DCF) on the studied materials under conditions of pH variation are presented in Figure 3.

With the increase of the initial pH of DCF solutions from 4 to 7, the adsorption capacity of all studied materials increases. At pH values equal to 8, it decreases and at even higher values, there is a new increase of the studied parameters, but this is due to the precipitation of DCF and not due to an adsorption process. The aspects presented show that the optimal pH value with which to reach the maximum adsorption capacity under the given working conditions is pH = 7. According to literature, diclofenac is a weak organic acid, and at pH = 7.4, more than 90% of it exists in ionized anionic form in the solutions [31,32]. For these reasons, using Mg_3_Al-LDH as adsorbent material, the optimum pH for DCF adsorption proved to be 7 due to electrostatic interaction between the positive charge of the LDH surface and DCF, which is found in ionized anions. This agrees with other results presented in literature [3,25]. This pH value was used in subsequent adsorption studies.

#### 2.2.2. Kinetic Studies

**Figure 4:**
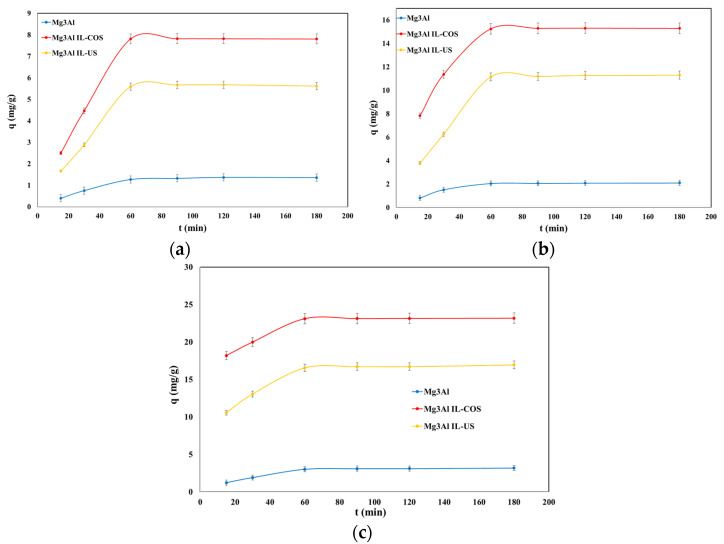
Dependence of adsorption capacity function of stirring time at DCF initial concentrations of C_0_ = 10 mg/L (**a**), 20 mg/L, (**b**) and 30 mg/L (**c**), and at T = 25 °C.

**Figure 5:**
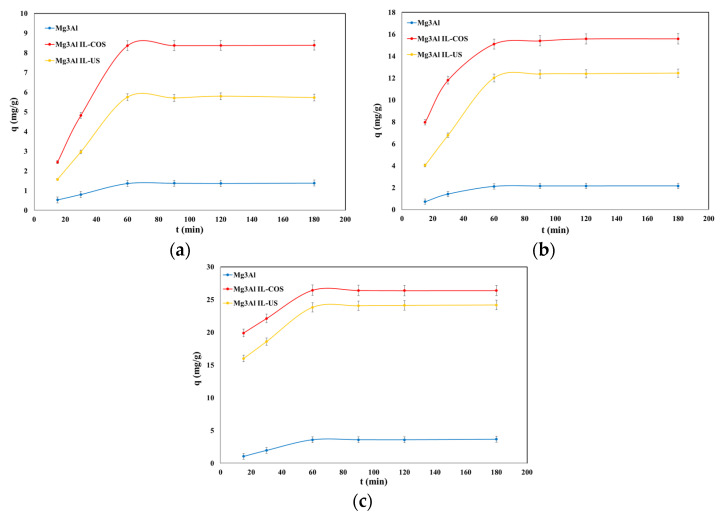
Dependence of adsorption capacity function of stirring time at DCF initial concentrations of C_0_ = 10 mg/L (**a**), 20 mg/L, (**b**) and 30 mg/L, (**c**) and at T = 40 °C.

**Figure 6:**
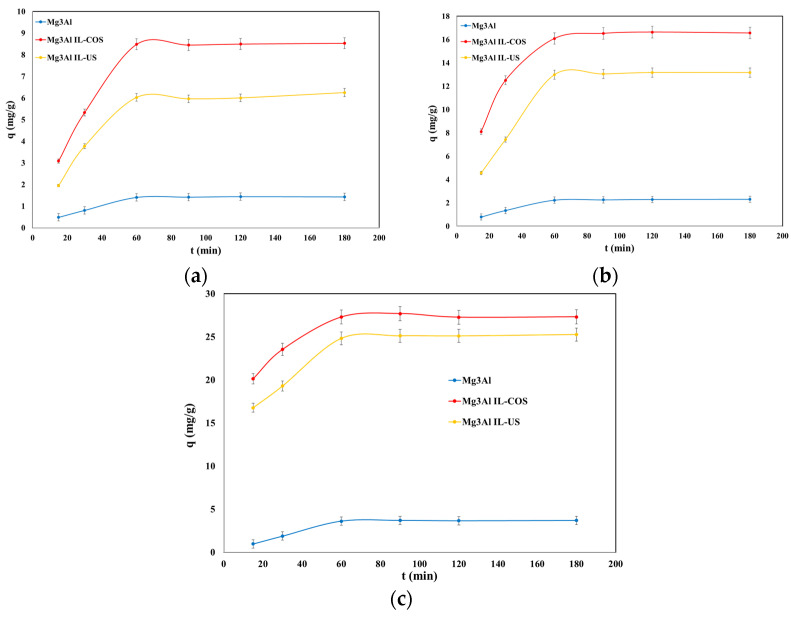
Dependence of adsorption capacity function of stirring time at DCF initial concentrations of C_0_ = 10 mg/L (**a**), 20 mg/L, (**b**) and 30 mg/L, (**c**) and at T = 55 °C.

Studies aimed the influence of time upon the adsorption process present a particular importance, as they provide information for determining the time required to reach the adsorption equilibrium; furthermore, the results can be used to understand the variables that influence the adsorption of the solute. Experimental data regarding the adsorption capacity dependence of the studied materials as a function of the contact time for 3 initial concentrations of DCF at 3 different temperatures are presented in Figure 4, Figure 5 and Figure 6.

For all the studied materials and for all the initial DCF concentrations, the adsorption capacity of the materials increases with the contact time increasing up to 60 min, after which it remains constant. At stirring times of 60 min, the adsorption capacity remains practically constant, probably due to occupying of the active sites from the adsorbent surface [26]. It is important to know the time required to achieve the equilibrium between adsorbent and adsorbate to estimate the time required for water treatment to extend the treatment process from the laboratory scale. The equilibrium between adsorbent and adsorbate is achieved quickly in 60 min. The behavior of the adsorption capacities of the studied materials’ function of time is similar, regardless of the initial concentration of DCF in the solution. Along with the increase of the initial concentration of DCF in aqueous solutions, the adsorption efficiency of the studied materials is intensified. The adsorption capacity showed a slight increase in tandem with an increasing working temperature. However, this is insignificant, and it is not economically justified to conduct the adsorption process at higher temperatures. The time of 60 min was considered the optimal contact time, as this was respectively the time required to reach the adsorption equilibrium.

Adsorption kinetics is an important feature that defines the efficiency of the adsorption process. To clarify the mechanism of diclofenac adsorption onto the studied materials and to identify the model that validates the experimental data, three kinetic models were used: pseudo-first-order kinetic model, and pseudo-second-order kinetic model, and intraparticle diffusion model [28,33].

For the modeling of the kinetic data, the integrated form of the Lagergren model was used, as described by the relation:

(1) $$\ln\left(q_{e}-q_{t}\right)={\mathrm{lnq}}_{t}-k_{1}\cdot t,$$

where:

q_t_—adsorption capacity of the adsorbent at time t, mg/g;
q_e_—adsorption capacity of the adsorbent at equilibrium, mg/g;
k_1_—adsorption rate constant, min^−1^;
t—stirring time, min.

From the linear dependencies ln(q_e_ − q_t_) as a function of t represented in Figure S1 (Supplementary Information), the velocity constants k_1_ were calculated, and the results are presented in Table 2, Table 3 and Table 4.

The pseudo-second-order kinetic model, proposed by Ho and McKay, can be expressed by the equation:

(2) $$\frac{t}{q_{t}}=\frac{1}{k_{2}\cdot q_{e}^{2}}+\frac{t}{q_{e}},$$

where:

k_2_—the rate constant of the pseudo-second-order kinetic adsorption model (g/mg/min)
q_e_—the amount of DCF adsorbed at equilibrium (mg/g);
q_t_—the amount of DCF adsorbed at time t (mg/g).

The graphical representations t/q_t_ as a function of time t allowed the evaluation of the rate constants k_2_ (from the slope of the lines), respectively, of the quantity of diclofenac adsorbed at equilibrium q_e_ (from the ordinate to the origin) (Figure S2 Supplementary Information).

The kinetic model of intraparticle diffusion is described by the Weber–Morris equation, which was used to identify the rate determinant stage if the intraparticle diffusion describes the kinetic process:

(3) qt=kint⋅t12+C,

where: k_int_ is the rate constant of the kinetic model of intraparticle diffusion (mg/g/min^1/2^).

The values of the k_int_ rate constants were estimated from the slopes of the q_t_ representations as a function of t^1/2^ (Figure S3 Supplementary Information).

According to Equation (3), the dependence of q_t_ as a function of t^1/2^ should lead to a straight line, the slope of which represents the rate constant k_int_ in which case the intra-particle diffusion is the determining speed step. If the line does not pass through the origin, the intraparticle diffusion may be accompanied by the film diffusion.

The experimental values and the calculated ones for the adsorption capacity at equilibrium, q_e_, and the values of the rate constants and of the regression coefficients for all the studied cases are presented in Table 2, Table 3 and Table 4.

According to the data presented in Table 2, Table 3 and Table 4, the application of the pseudo-first-order kinetic model led to large differences between the values of the equilibrium adsorption capacity determined experimentally and those obtained by modeling. This observation, correlated with the lower values of the regression coefficients, led to the conclusion that the pseudo-first-order kinetic model does not describe the adsorption process of diclofenac onto the studied materials.

The linear dependencies t/q_t_ as a function of t (Figure S2), obtained by applying the kinetic model of pseudo-second-order, indicate a good concordance of this model with the experimental data. Moreover, the correlation coefficients resulting from the linear representations have values higher than 0.98 for all the studied situations. Also, the values of the adsorption capacity obtained experimentally are close to the values obtained from the graphical representations for all the studied materials at all 3 initial concentrations of DCF from aqueous solutions and at all 3 temperatures. Therefore, the DCF adsorption process on the studied materials is faithfully described by the pseudo-second-order kinetic model. The adsorption efficiency is much higher for the functionalized Mg_3_Al samples.

From the q_e_ dependencies as a function of t^1/2^ (Figure S3), the DCF adsorption on the studied materials has a complex mechanism which takes place in two stages corresponding to the two levels in the diagrams. The first stage corresponds to an instant adsorption process, characterized by the diffusion of the solute at the external surface of the adsorbent. The second level, with a lower slope, can be attributed to the adsorption process that takes place gradually at the pores of the adsorbent materials and corresponds to the rate determining the speed [34].

The best fit of DCF adsorption data with the pseudo-second-order kinetic model indicates that the adsorption process correspond to a chemisorption as the rate-limiting mechanism, which occurs by electron sharing or exchange between DCF and active sites/functional groups of the studied adsorbent materials [27].

#### 2.2.3. Thermodynamic Studies

To assess the nature of the adsorption process of DCF onto the studied materials, the thermodynamic parameters, such as standard enthalpy (∆H^0^), entropy (∆S^0^), and Gibbs free energy (∆G^0^), were evaluated using the following equations:

ΔG^0^ = −RTlnK_d_, (4)

ΔG^0^ = ΔH^0^ − TΔS^0^, (5)

(6) $${\mathrm{lnK}}_{d}=\frac{\Delta S^{0}}{R}-\frac{\Delta H^{0}}{\mathrm{RT}},$$

(7) $$K_{d}=\frac{q_{e}}{C_{e}}$$

where: ΔG is Gibbs free energy (KJ/mol), ΔS is the entropy and heat of adsorption (J/mol K), ΔH is enthalpy (kJ/mol), T is the absolute temperature (K), R is universal gas constant (8.314 J/(mol∙K)), and K_d_ is the distribution coefficient.

**Figure 7:**
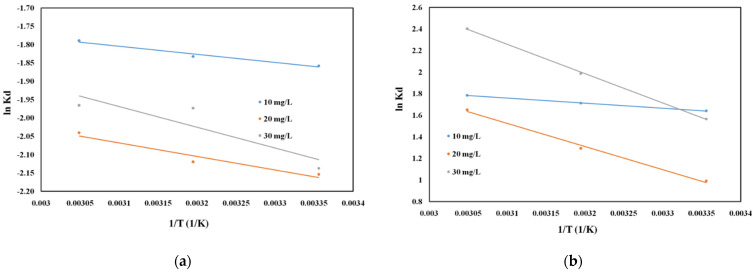

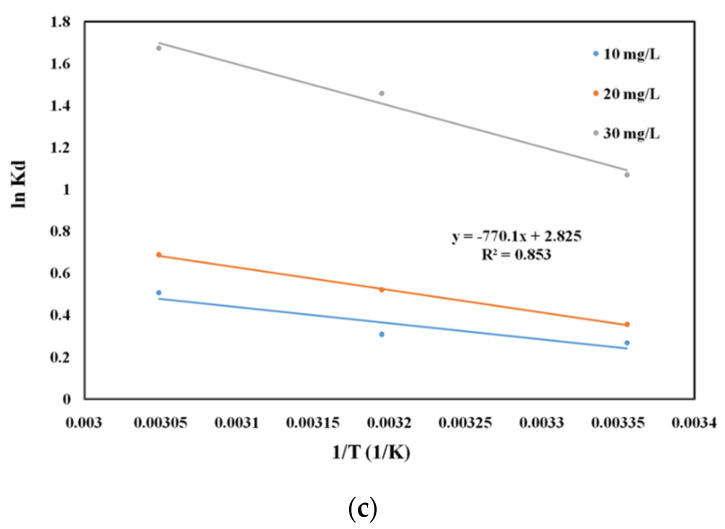
Thermodynamic plot of ln Kd vs. 1/T for DCF adsorption onto studied materials ((**a**) Mg_3_Al, (**b**) Mg_3_Al IL-COS, and (**c**) Mg_3_Al IL-US) at three different initial concentration of DCF.

The thermodynamic variables ΔH^0^ and ΔS^0^ were obtained from the slope and intercept of Von’t Hoff plots of lnK_d_ versus 1/T (Figure 7), and the resulting values, together with the Gibbs free energy ΔG^0^ calculated with Equation (5) and the correlation coefficients, are presented in Table 5.

The positive value of ΔH^0^ indicates that the adsorption process of DCF onto the studied materials is endothermic in nature. In case of the functionalized samples (Mg_3_Al IL-COS and Mg_3_Al IL-US), the feasibility and spontaneity of the adsorption process was confirmed by the obtained negative values of ΔG^0^, and these values are more negatively with the temperature increasing and the initial concentration of DCF increasing. This suggests that at higher concentrations of DCF in the solution, more active groups are available in the solution, assuring a better contact with the adsorbent surface and leading to a greater adsorption efficiency. The positive value of ΔS^0^ suggested an increase in randomness at the solid/liquid interface during adsorption of DCF onto the functionalized samples. In case of raw Mg_3_Al, due to the absence of the functional group given by the studied IL from the adsorbent surface, it diminished the randomness at the solid/liquid interface, a fact that was also suggested by the obtained negative value ΔS^0^.

#### 2.2.4. Equilibrium Studies

Equilibrium studies provide information on the maximum adsorption capacity and affinity of the materials used as adsorbents in relation to the target pollutants. Isotherm studies are essential to interpret the interaction between the adsorbent and adsorbate. The modeling of experimental data allows the identification of the process mechanism and the theoretical evaluation of the adsorption capacities at equilibrium. In this paper, the nonlinear equation of Langmuir, Freundlich, and Sips isotherms were used for modeling the experimental data.

Langmuir isotherm supposes that the adsorption of the target pollutant takes place as a complete monolayer coverage of the adsorbent surface, and its equation is given as:

(8) $$q_{e}=q_{\mathrm{mL}}\frac{K_{L}C_{e}}{{1+K}_{L}C_{e}},$$

where:

q_e_—amount of DCF adsorbed at equilibrium (mg/g)
C_e_—equilibrium concentration of DCF in solution (mg/L)
q_m_—maximum adsorption capacity in Langmuir model (mg/g)
K_L_—Langmuir constant (L/mg).

In contrast the Freundlich isotherm supposes that the adsorption occurs in multilayers. The nonlinear form of Freundlich isotherm is given by Equation (9):

(9) $$q_{e}=K_{F}\cdot C_{e}^{1/n},$$

where:

K_F_—Freundlich isotherm constant (mg^1−1/n^ L^1/n^/g)
n—Freundlich exponent.

To predict the heterogenous adsorption systems and localized adsorption without adsorbate–adsorbent interaction, a combined form of Langmuir and Freundlich isotherm, the Sips model, is used. This model is given by the next equation:

(10) $$q_{e}=q_{\mathrm{mS}}\frac{K_{S}C_{e}^{m}}{{1+K}_{S}C_{e}^{m}},$$

where:

q_mS_—maximum adsorption capacity in Sips model (mg/g)
K_S_—Sips isotherm constant (L^m^/mg^m^)
m—Sips exponent.

**Figure 8:**
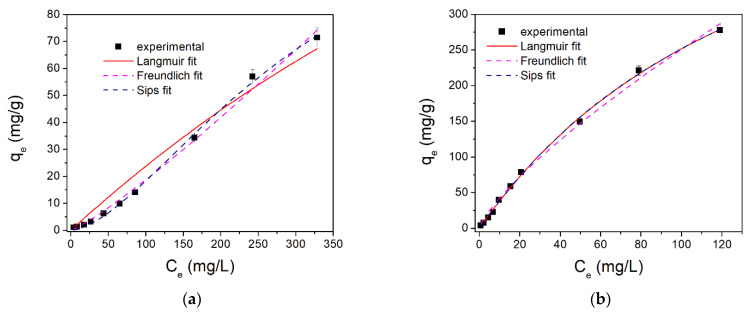

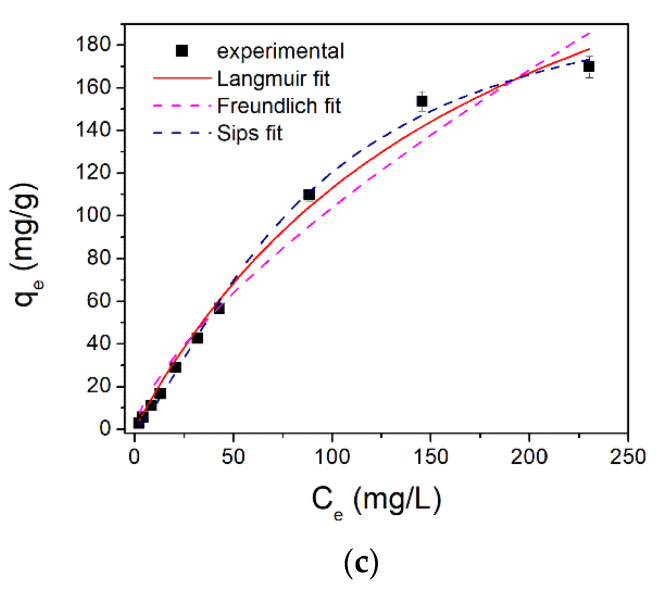
Nonlinear fit of DCF adsorption onto: (**a**) Mg_3_Al, (**b**) Mg_3_Al IL-COS, and (**c**) Mg_3_Al IL-US.

The graphical representation of the studied isotherm for the DCF adsorption onto the studied materials is presented in Figure 8, and the obtained parameters are summarized in Table 6.

The functionalization of adsorbent materials with the studied ionic liquid leads to an increase in their adsorbent efficiency, which means that the functional groups from the ionic liquid contribute to the process of DCF removal from aqueous solutions. Through ultrasonic functionalization of the LDH, the adsorption capacity of the obtained Mg_3_Al IL-US material increases by two-times greater than the raw Mg_3_Al material. The sample obtained by cosynthesis (Mg_3_Al IL-COS) develops a maximum adsorption capacity five-times higher than the capacity developed by the raw LDH. In this case, with IL being between the LDH layers, as was observed from the RX and SEM analysis of the samples, the DCF adsorption occurs due to a synergetic effect between the functional groups of IL and the porosity of the LDH surface. By functionalizing the LDH with the studied IL by cosynthesis, the cover of the LDH surface in the form of a film is avoided, which leads to a decrease in the porosity of the material, and implicitly, the decrease in adsorption in the LDH pores of DCF, as it happened in the case of samples obtained by ultrasound. Thus, in this case, both the functional groups of IL and the surface of LDH contribute to the removal of DCF from aqueous solutions, obtaining the highest value of the maximum adsorption capacity.

Comparing the parameters of the used isotherm for the experimental data modeling, the DCF adsorption onto the studied materials is best described by the Sips model. In this case, we obtained the correlation coefficients close to unity and maximum adsorption capacity calculated from the model close with those experimentally obtained. This indicates that the DCF adsorption onto the studied materials occurs as a heterogeneous process, not as a monolayer covering.

**Figure 9:**
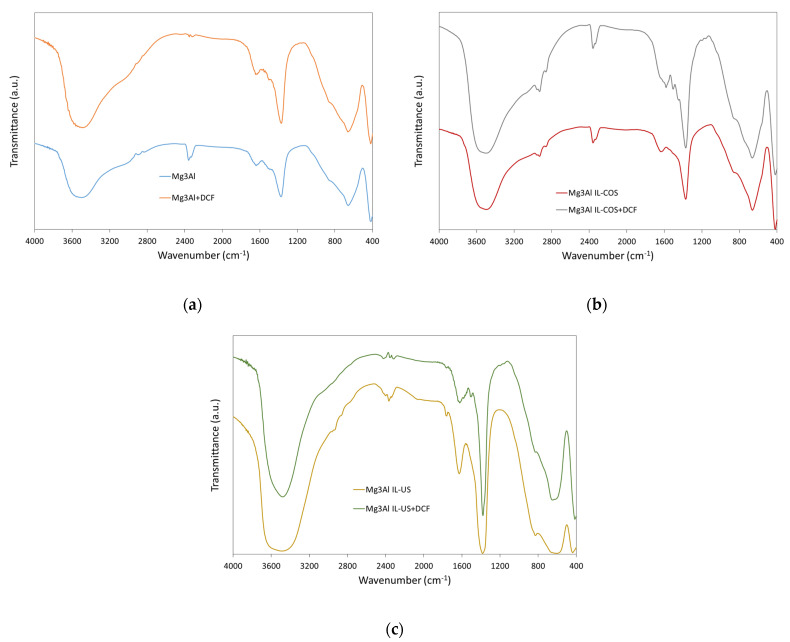
Fourier-transform infrared spectroscopy (FTIR) spectra of: (**a**) Mg_3_Al, (**b**) Mg_3_Al IL-COS, and (**c**) Mg_3_Al IL-US before and after DCF adsorption.

Figure 9 presents the FTIR spectra of adsorbents before and after DCF adsorption. The FTIR spectrum of Mg_3_Al (Figure 9a) exhibits characteristic peaks of layered double hydroxide with carbonate as interlayer anion: the broad and strong absorption band in the range of 3600–3200 cm^−1^ is due to the O-H stretching vibration of the surface and interlayer water molecules. The broad shoulder at 3050 cm^−1^ could been attributed to hydrogen bounding between water and carbonate anions in the interlayer galleries [35]. The adsorption band with maximum at 1640 cm^−1^ is due to the water bending vibration of the interlayer water molecules. The absorption band located around 1500 cm^−1^ is due to the stretching vibrations of carbonate anions. The presence of a multiple band between 1200–1600 cm^−1^, and the lack of even a single intense band at 1383 cm^−1^ indicates the lowering of the symmetry of carbonate anions from the planar D_3h_ to the C_2ν_ symmetry. Weakly resolved shoulder present at 850 cm^−1^ could be attributed to bending vibrations of carbonates. In addition, the spectrum contains absorption bands at 650 cm^−1^ (bending vibrations of carbonate bonds) and 420 cm^−1^ (vibrations of Mg-OH, Al-OH and Zn-OH bonds from octahedral networks of layered double hydroxide) [36].

The adsorbents prepared by cosynthesis (Mg_3_Al IL-COS, Figure 9b) and by ultrasonication (Mg_3_Al IL-US, Figure 9c) present, besides the characteristic absorption bans of layered double hydroxide, a doublet of shoulders (at 2860 and 2930 cm^−1^) that overlap to the shoulder assigned to hydrogen bonding in the LDH, and they could be assigned to the ν_3_ vibrations of -CH_3_ group in the IL [22]. This doublet is more evident in the FTIR spectrum of Mg_3_Al IL-COS sample, and it proves the presence of IL in the layered double hydroxide structure.

Sodium diclofenac spectrum exhibits some characteristic IR-absorption bands: one peak at about 1600 cm^−1^, attributed to the C=C bonds in benzene ring, two adsorption bands located at 1576 and 1507 cm^−1^ due to C=O stretching and C=C stretching of carboxyl group, and two peaks located at 1306 and 1283 cm^−1^, attributed to C-N stretching vibrations [37]. After DCF adsorption, in the adsorbents’ spectra, the vibration bands due to the carboxyl group from the DCF structure appear. The weak shoulders at 1500 and 1575 cm^−1^, as observed in the Mg_3_Al spectrum after DCF adsorption (Figure 9a), at 1506 and 1579 cm^−1^, as observed in the Mg_3_Al IL-COS spectrum (Figure 9b), and at 1504 and 1581 cm^−1^, as observed in the Mg_3_Al IL-US spectrum (Figure 9c), could be attributed to the C=O stretching and C=C stretching of carboxyl group from diclofenac. This absorption band overlaps with the band attributed to stretching vibrations of carbonate anion; it is presumed that the carboxyl group replaced some carbonate anions placed onto the positive surface of layered double hydroxides. The higher the amount of DCF adsorbed, as the equilibrium studies reveal, the more visible and intense this IR-absorption bands are.

Comparing the maximum adsorption capacity developed by the studied materials with the adsorption capacity, as reported in literature and developed by other materials in the removal process of DCF from aqueous solutions (Table 7), we obtained through functionalization of Mg_3_Al with methyl trialkyl ammonium chloride, especially through cosynthesis method, an efficient and high-performance adsorbent that could be used in the treatment process of waters with pharmaceutical content.

## 3. Materials and Methods

### 3.1. Materials 

Commercially available analytical grade reagents (i.e., magnesium nitrate hexahydrate, aluminum nitrate nonahydrate, sodium hydroxide, sodium carbonate, hydrochloric acid) were purchased from Merck (Darmstadt, Germany). Methyl trialkyl ammonium chloride (IL) and sodium diclofenac (DCF) were purchased from Sigma-Aldrich (Steinheim, Germany), and were used as received. 

### 3.2. Adsorbent Obtaining and Characterization

Mg_3_Al-LDH was synthetized by coprecipitation method at low supra-saturation [39]. As metal precursor were used nitrate salts (Mg(NO_3_)_2_·6H_2_O and Al(NO_3_)_3_·9H_2_O). A mixed solution of 1 M concentration, containing the metal ions in the ratio, Mg:Al = 3:1, was added drop by drop into 100 mL, 1 M Na_2_CO_3_ solution. The suspension was kept continuous under vigorous stirring, maintaining the pH around 10.5 value, with the help of 2M NaOH solution. After 24 h of maturation at 70 °C, the mass reaction was filtered and washed until pH = 7, using distilled water. The obtained product was dried for 24 h at 70 °C, and then crushed and sieved to a dimension lower than 90 µm. Further in the manuscript for this sample, Mg_3_Al symbol will be used.

The functionalization of Mg_3_Al with methyl trialkyl ammonium chloride was made using two methods. The first one assures the distribution of the studied IL onto the surface of the synthesized LDH because is used the ultrasound method [12,19,20]. For this purpose, a well-defined quantity of Mg_3_Al was treated with a well-established quantity of IL (in finality, to have 10% of IL in the adsorbent mass), which previously was dissolved in acetone. The obtained suspension was subject to 10 min of ultrasonication, using an ultrasound bath. After functionalization, the solvent was evaporated at 50 °C for 24 h. Further in the manuscript for this sample, Mg_3_Al IL-US symbol will be used. The second method of functionalization implies the intercalation of the ILs between the LDH interlayers [12]. In this case, the cosynthesis method was used, as was described above for the obtaining of Mg_3_Al, with the exception that, in this case, instead of Na_2_CO_3_ solution, the IL dissolved in acetone was used. Further in the manuscript for this sample, Mg_3_Al IL-COS symbol will be used. To prove that Mg_3_Al was synthesized and its functionalization with methyl trialkyl ammonium chloride occurred, the obtained adsorbent materials were characterized through XRD and SEM analysis. A Rigaku Ultima IV X-ray diffractometer (Rigaku Analytical Devices Inc., Wilmington, MA, USA) (40 kV, 40 mA) with Cu_Kα_ radiation was used for the recording of the X-ray diffractograms. SEM images were recorded using a Quanta FEG 250 microscope (FEI Company, Hillsboro, OR, USA), equipped with an EDAX/ZAF quantifier. The FTIR spectra of the samples prepared as KBr pellets were recorded by using a Shimadzu IRPrestige-21 FTIR spectrophotometer (Shimadzu Corporation, Kyoto, Japan) in the range 400–4000 cm^−1^ and a nominal resolution of 4 cm^−1^.

### 3.3. Adsorption Studies

To evaluate the adsorption performance of the obtained materials, the synthesized and characterized samples were used as adsorbent materials in the removal process of DCF from aqueous solutions. Their efficiency in the DCF adsorption process was studied according to a series of physico-chemical parameters: the initial pH of the aqueous solution, the stirring time of the reaction mass, the temperature, and the initial concentration of DCF in the solution, respectively.

In the first step, the influence of pH upon the adsorption efficiency developed by the studied materials was studied. In this sense, the adsorption studies were conducted at initial pH values of the mass reaction, ranged between 4–10. Outside this range, the layered double hydroxides solubilize. The other physico-chemical parameters studied were kept constant (initial concentration of DCF, C_0_ = 10 mg/L; stirring time, t = 60 min; temperature T = 25 °C).

Studies regarding the influence of agitation time on the adsorbent capacity developed by the studied materials in the removal process of DCF were performed in the range of 15–180 min, using 3 different initial concentrations of DCF solutions (10, 20, and 30 mg DCF/L) at 3 temperatures (25, 40 and 55 °C), the initial pH of the solutions being around pH = 7.

To determine the maximum adsorption capacity of the studied materials developed in the removal process of DCF from aqueous solutions, studies were performed by varying the initial concentration of DCF solutions (5–400 mg/L), maintaining the other parameters at the optimal values previously determined: pH = 7, t = 60 min, T = 25 °C. In all adsorption studies, the solid–liquid ratio was (adsorbent material:solution containing DCF) S:L = 1:1 (0.025 g adsorbent material in 25 mL solution containing DCF).

The adsorption capacity of the studied materials was determined using the following equation:

(11) $$q_{e}=\frac{\left(C_{0}{-C}_{e}\right)V}{m},$$

where:

q_e_—equilibrium capacity developed by adsorbent materials, mg DCF/g adsorbent material.
C_0_—initial concentration of DCF in aqueous solutions, mg/L.
C_e_—equilibrium concentration of DCF, mg/L.
V—volume of the solution containing DCF used in the adsorption process, L.
m—mass of adsorbent material used in the adsorption process, g.

## 4. Conclusions

The present paper highlights the benefits brought by ionic liquids in the process of obtaining efficient adsorbent materials via its functionalization on layered double hydroxide solid support. As a solid support, we used Mg_3_Al LDH, which was functionalized with methyl trialkyl ammonium chloride by ultrasound and cosynthesis methods. The RX and SEM analysis performed on the obtained adsorbent materials proved that the functionalization occurred. The adsorption efficiency of the obtained materials was tested in the removal process of diclofenac from aqueous solutions. In all cases, the equilibrium between the adsorbent and adsorbate was achieved in 60 min, and the maximum removal efficiency was obtained at an initial DCF solution pH of around 7. The DCF adsorption onto the studied materials is best described by the pseudo-second-order kinetic model. The samples functionalized with the studied IL showed a better performance than that of raw Mg_3_Al. Correlating the results from the adsorbent characterization section with the results from the equilibrium and thermodynamic studies, the beneficial role of the IL from the adsorbent structure in the adsorption process of DCF from aqueous solutions is highlighted. Its presence contributes to the (1) disturbance of the well-ordered structure of Mg_3_Al, which leads to an increasing in the active sites from the adsorbent surface; (2) bringing more functional groups into the adsorbent structure, and providing a synergic effect in the adsorption process; (3) due to its functional group, assuring some electrostatic interaction with the target pollutant, and determining this random and heterogeneous adsorption of DCF from aqueous solutions.

## Figures and Tables

**Table 1:** Unit cell parameters for synthesized samples.

Sample	a (Å)	c (Å)	D (nm)
Mg_3_Al	3.0730	23.58	4.86
Mg_3_Al IL-COS	3.0725	24.39	3.38
Mg_3_Al IL-US	3.0710	23.53	4.45

**Table 2:** Kinetic parameters resulted from application of kinetic models on adsorption of DCF onto studied materials at different initial concentrations of DCF and at T = 25 °C.

Adsorbent	q_e_ Exp (mg/g)	Pseudo-First-Order			Pseudo-Second-Order			Intraparticle Diffusion		
		q_e_ Calc (mg/g)	k_1_ (min^−1^)	R^2^	q_e_ Calc (mg/g)	k_2_·10^−3^ (min/(mg/g))	R^2^	k_int_ (mg/g min^−1/2^)	C	R^2^
**C_0_ = 10 mg/L**										
Mg_3_Al	1.38	0.845	0.0250	0.8045	1.71	16.3	0.9650	0.102	0.209	0.7741
Mg_3_Al IL-COS	8.02	2.89	0.0197	0.5748	9.48	3.67	0.9665	0.556	1.65	0.7146
Mg_3_Al IL-US	6.00	2.70	0.0149	0.5821	7.11	3.92	0.9511	0.429	0.873	0.7231
**C_0_ = 20 mg/L**										
Mg_3_Al	2.29	0.934	0.0112	0.6465	2.37	23.0	0.9866	0.123	0.722	0.6941
Mg_3_Al IL-COS	15.5	3.78	0.0217	0.6082	16.6	5.15	0.9945	0.744	7.07	0.6993
Mg_3_Al IL-US	11.9	5.23	0.0162	0.6440	13.7	2.54	0.9711	0.800	2.36	0.7328
**C_0_ = 30 mg/L**										
Mg_3_Al	3.38	1.64	0.0141	0.7576	3.69	11.5	0.9876	0.204	0.852	0.7689
Mg_3_Al IL-COS	23.4	3.07	0.0197	0.6411	23.9	10.4	0.9994	0.524	17.4	0.7246
Mg_3_Al IL-US	17.1	4.96	0.0205	0.8069	17.9	6.33	0.9984	0.653	9.42	0.7502

**Table 3:** Kinetic parameters resulting from application of kinetic models on adsorption of DCF onto studied materials at different initial concentrations of DCF and at T = 40 °C.

Adsorbent	q_e_ Exp (mg/g)	Pseudo-First-Order			Pseudo-Second-Order			Intraparticle Diffusion		
		q_e_ Calc (mg/g)	k_1_ (min^−1^)	R^2^	q_e_ Calc (mg/g)	k_2_·10^−3^ (min/(mg/g))	R^2^	k_int_ (mg/g min^−1/2^)	C	R^2^
**C_0_ = 10 mg/L**										
Mg_3_Al	1.59	0.771	0.0096	0.6149	1.79	26.1	0.9810	0.218	−0.342	0.9908
Mg_3_Al IL-COS	8.58	3.25	0.0207	0.6035	11.1	3.07	0.9579	1.53	−3.51	0.9998
Mg_3_Al IL-US	5.95	2.44	0.0185	0.5729	7.36	3.59	0.9429	1.09	−2.77	0.9918
**C_0_ = 20 mg/L**										
Mg_3_Al	2.35	1.27	0.0121	0.6278	2.53	16.5	0.9758	0.356	−0.613	0.9902
Mg_3_Al IL-COS	15.8	5.41	0.0255	0.7992	16.9	4.92	0.9962	1.82	1.26	0.9795
Mg_3_Al IL-US	12.7	6.24	0.0237	0.7595	15.2	2.13	0.9701	2.08	−4.23	0.9939
**C_0_ = 30 mg/L**										
Mg_3_Al	3.87	1.89	0.0153	0.6699	4.57	6.32	0.9605	0.656	−1.53	0.9960
Mg_3_Al IL-COS	26.6	3.24	0.0206	0.5313	27.3	7.84	0.9990	1.70	13.1	0.9928
Mg_3_Al IL-US	34.4	5.97	0.0232	0.7534	25.5	5.01	0.9981	2.04	7.87	0.9912

**Table 4:** Kinetic parameters resulting from application of kinetic models on adsorption of DCF onto studied materials at different initial concentrations of DCF and at T = 55 °C.

Adsorbent	q_e_ Exp (mg/g)	Pseudo-First-Order			Pseudo-Second-Order			Intraparticle Diffusion		
		q_e_ Calc (mg/g)	k_1_ (min^−1^)	R^2^	q_e_ Calc (mg/g)	k_2_·10^−3^ (min/(mg/g))	R^2^	k_int_ (mg/g min^−1/2^)	C	R^2^
**C_0_ = 10 mg/L**										
Mg_3_Al	1.64	0.834	0.0103	0.6260	1.83	20.4	0.9720	0.241	−0.475	0.9946
Mg_3_Al IL-COS	8.73	3.22	0.0198	0.6406	11.1	4.35	0.9802	1.39	−2.31	1.000
Mg_3_Al IL-US	6.45	3.26	0.0174	0.7714	7.48	4.57	0.9754	1.05	−2.06	0.9987
**C_0_ = 20 mg/L**										
Mg_3_Al	2.51	1.23	0.0128	0.6927	2.76	13.1	0.9780	0.375	−0.685	0.9991
Mg_3_Al IL-COS	16.8	5.38	0.0220	0.7210	19.1	3.91	0.9947	2.03	0.675	0.9751
Mg_3_Al IL-US	13.4	5.63	0.0235	0.7014	15.8	2.31	0.9742	2.19	−4.18	0.9933
**C_0_ = 30 mg/L**										
Mg_3_Al	3.88	1.02	0.0162	0.6330	4.78	5.16	0.9404	0.687	−1.76	0.9935
Mg_3_Al IL-COS	27.9	3.76	0.0153	0.4527	28.2	8.11	0.9989	1.83	13.2	0.9952
Mg_3_Al IL-US	25.5	6.59	0.0231	0.7785	26.6	4.72	0.9981	2.10	8.31	0.9872

**Table 5:** Thermodynamic parameters of DCF adsorption onto studied materials.

Adsorbent	C_0_ (mg/L)	ΔH (KJ/mol)	ΔS (J/mol·K)	ΔG (KJ/mol)			R^2^
				298 (K)	313 (K)	328 (K)	
Mg_3_Al	10	1.83	−9.31	4.60	4.74	4.88	0.9697
	20	3.06	−7.69	5.35	5.46	5.58	0.9352
	30	4.69	−1.81	5.22	5.25	5.28	0.8038
Mg_3_Al IL-COS	10	3.93	26.8	−4.08	−4.48	−4.88	0.9992
	20	17.8	63.9	−1.23	−2.19	−3.14	0.9944
	30	22.6	88.9	−3.86	−5.20	−6.53	0.9995
Mg_3_Al IL-US	10	6.40	23.4	−0.597	−0.949	−1.30	0.8538
	20	8.95	32.9	−0.881	−1.37	−1.87	0.9989
	30	16.4	64.1	−2.70	−3.66	−4.63	0.9819

**Table 6:** Langmuir, Freundlich, and Sips parameters for DCF adsorption onto studied materials.

Model	Parameter	Mg_3_Al	Mg_3_Al IL-COS	Mg_3_Al IL-US
Langmuir	q_mL_ (mg/g) K_L_ (L/mg) R^2^	334 0.00077 0.9712	648 0.00635 0.9987	320 0.00547 0.9905
Freundlich	K_F_ (mg^1−1/n^ L^1/n^/g) 1/n R^2^	0.0922 0.155 0.9935	7.25 0.770 0.9948	4.16 0.698 0.9700
Sips	q_mS_ (mg/g) K_S_ (L^m^/mg^m^) m R^2^	143 0.00008 1.62 0.9982	628 0.00635 1.01 0.9987	217 0.00207 1.39 0.9974

**Table 7:** Comparison between adsorption capacities of similar adsorbents developed in treatment processes of aqueous solutions containing DCF.

Adsorbent Materials	pH	q_m_ (mg/g)	References
MgAl/layered double hydroxide supported on *Syagrus coronata* biochar	5.65	168	[3]
Organobentonite with hexadecyltrimethylammonium (OBHDTMA)	7.0	388	[25]
GAC	4.0	6.847	[27]
Alginate/Carbon-based Films	3.0	29.9	[28]
MgAl-CO_3_	10	562.4	[33]
K10 montmorillonite intercalated with cetyltrimethyl-ammonium bromide cations	7.0	55.46	[38]
Zn Al—LDH	7.0	94.32	
ZnAl—LDH calcinated	7.0	737.02	
Mg_3_Al	7.0	143	Present paper
Mg_3_Al IL-US	7.0	217	
Mg_3_Al IL-COS	7.0	628	

## Data Availability

The data are available on request from the corresponding authors. Supplementary Materials are included.

## Supplementary Materials

The following are available online. Figure S1: Linear representation of the pseudo-first-order kinetic model for DCF adsorption onto the studied materials, Figure S2: Linear representation of the pseudo-second-order kinetic model for DCF adsorption onto the studied materials, Figure S3: Representation of the intraparticle diffusion model for DCF adsorption onto the studied materials.

## Sample Availability

Samples of the compounds are available from the authors.
