# Supplementary material for: Development of New Efficient Adsorbent by Functionalization of Mg3Al-LDH with Methyl Trialkyl Ammonium Chloride Ionic Liquid
Source: Molecules. 2021 Dec 5;26(23):7384. doi: 10.3390/molecules26237384 (PMC8659049; doi:10.3390/molecules26237384)
Supplement: Supplementary file 1 [file molecules-26-07384-s001.zip › molecules-1463473-supplementary.pdf]

Supplementary Information

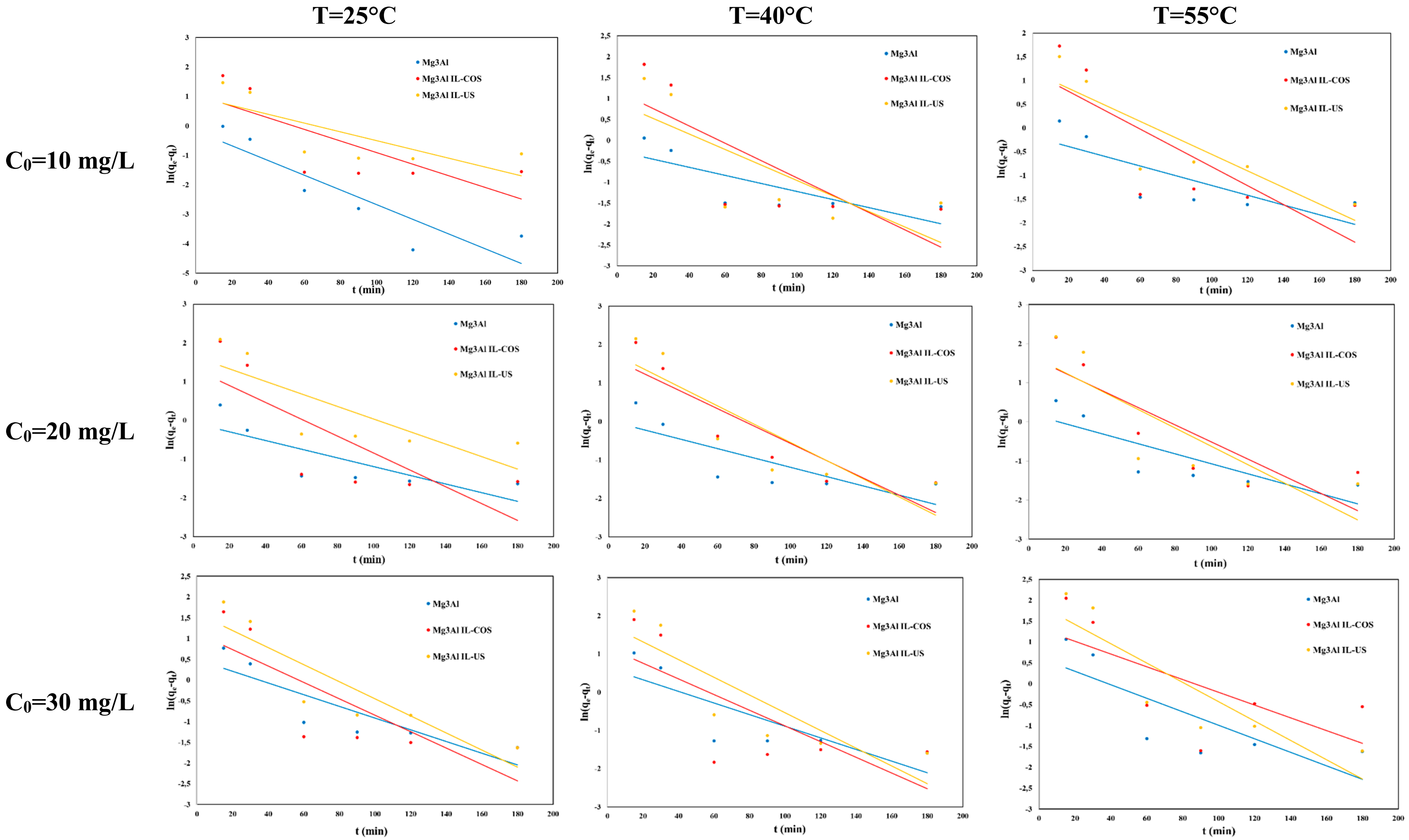

Figure S1. Linear representation of the pseudo-first order kinetic model for DCF adsorption onto the studied materials

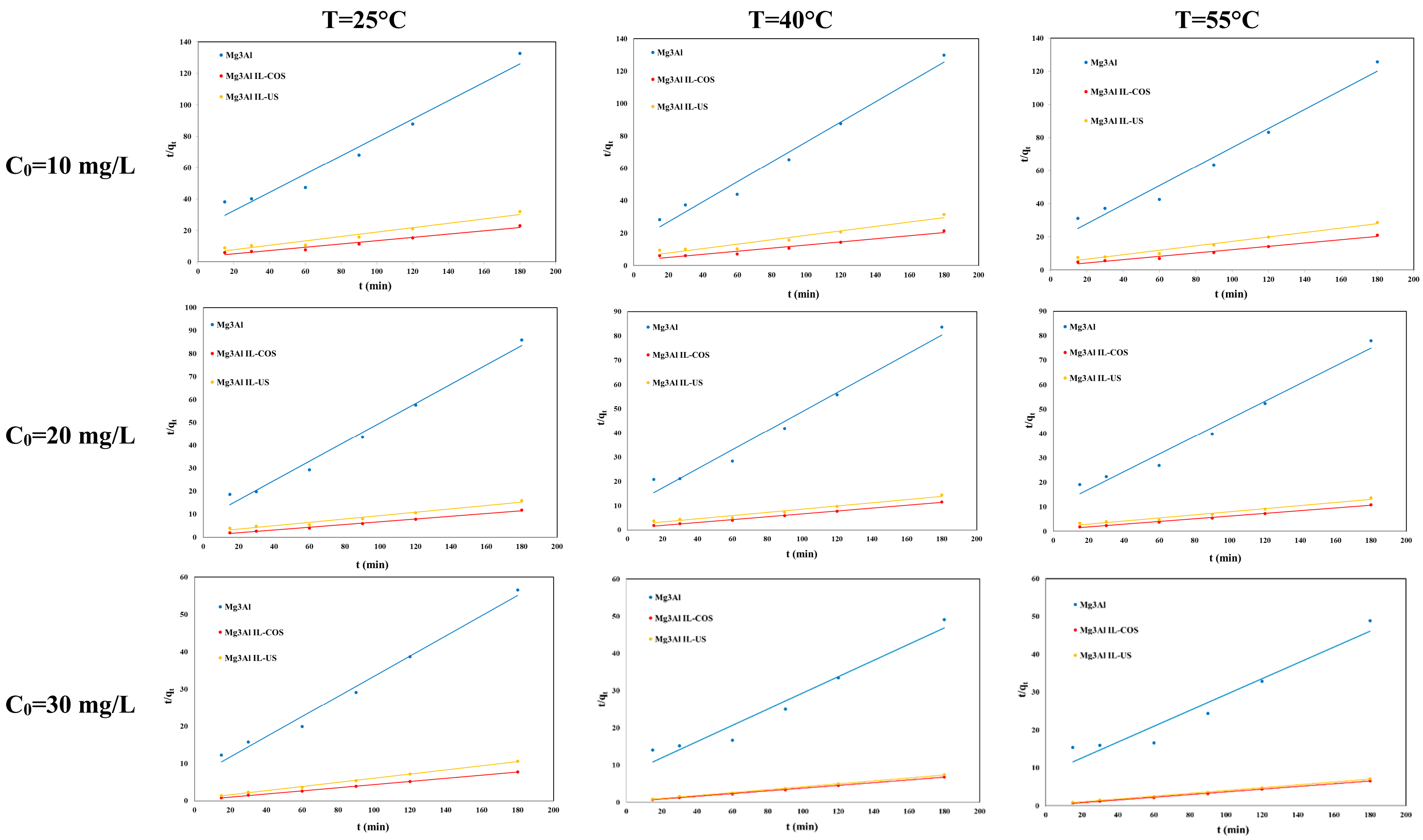

Figure S2. Linear representation of the pseudo-second order kinetic model for DCF adsorption onto the studied materials

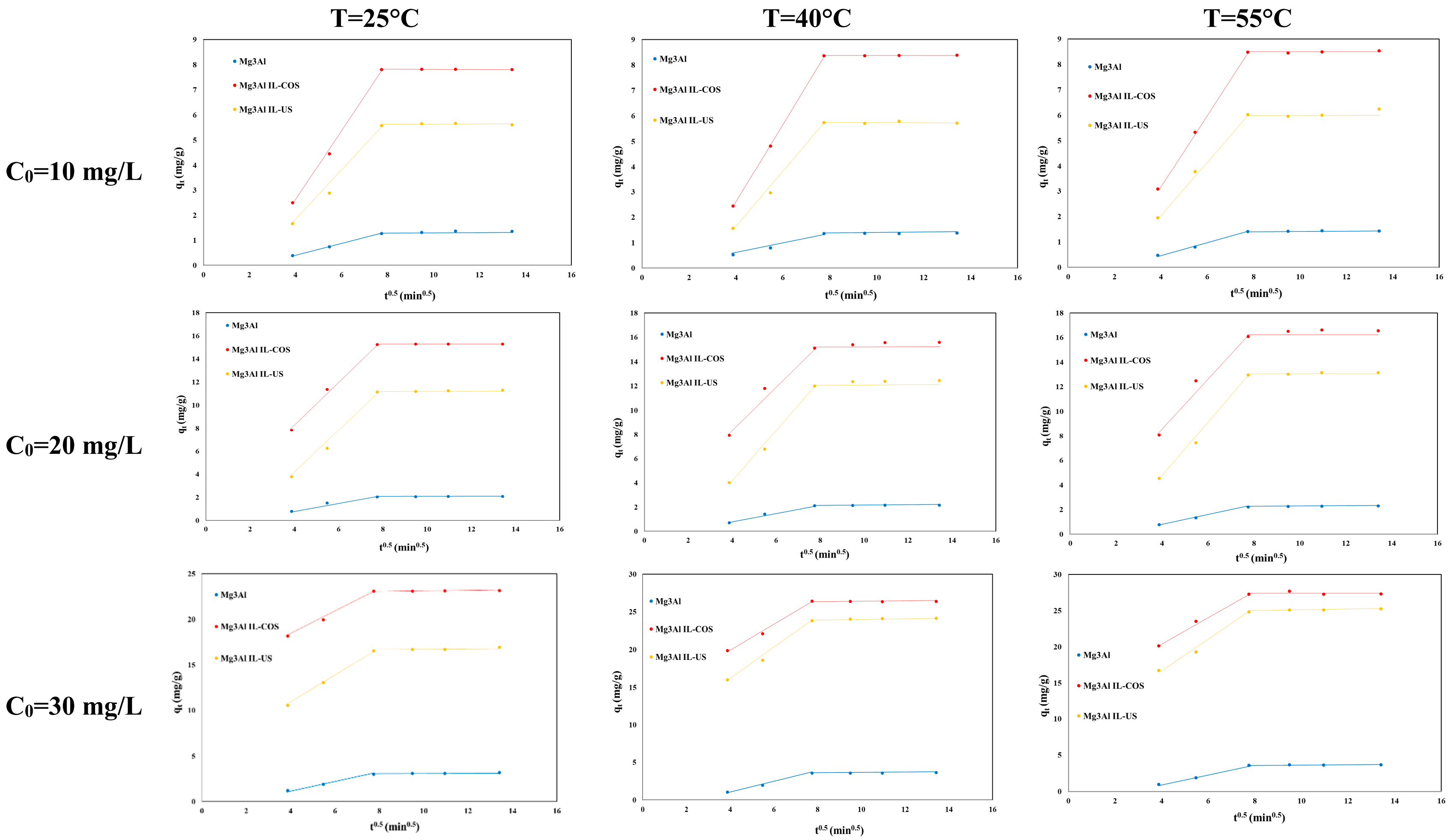

Figure S3. Representation of the intraparticle diffusion model for DCF adsorption onto the studied materials
